# Characterization and Functional Importance of Two Glycoside Hydrolase Family 16 Genes from the Rice White Tip Nematode *Aphelenchoides besseyi*

**DOI:** 10.3390/ani11020374

**Published:** 2021-02-02

**Authors:** Hui Feng, Dongmei Zhou, Paul Daly, Xiaoyu Wang, Lihui Wei

**Affiliations:** Institute of Plant Protection, Jiangsu Academy of Agricultural Sciences, 210014 Nanjing, China; fenghui@jaas.ac.cn (H.F.); luoyuerzhou@126.com (D.Z.); paul.daly@jaas.ac.cn (P.D.); wxyz999@126.com (X.W.)

**Keywords:** glycoside hydrolase family 16, *Aphelenchoides besseyi*, fungal feeding, RNA interference

## Abstract

**Simple Summary:**

The rice white tip nematode *Aphelenchoides besseyi* is a plant parasite but can also feed on fungi if this alternative nutrient source is available. Glucans are a major nutrient source found in fungi, and β-linked glucans from fungi can be hydrolyzed by β-glucanases from the glycoside hydrolase family 16 (GH16). The GH16 family is abundant in *A. besseyi,* but their functions have not been well studied, prompting the analysis of two GH16 members (AbGH16-1 and AbGH16-2). AbGH16-1 and AbGH16-2 are most similar to GH16s from fungi and probably originated from fungi via a horizontal gene transfer event. These two genes are important for feeding on fungi: transcript levels increased when cultured with the fungus *Botrytis cinerea*, and the purified AbGH16-1 and AbGH16-2 proteins inhibited the growth of *B. cinerea*. When AbGH16-1 and AbGH16-2 expression was silenced, the reproduction ability of *A. besseyi* was reduced. These findings have proved for the first time that GH16s contribute to the feeding and reproduction of *A. besseyi*, which thus provides novel insights into how plant-parasitic nematodes can obtain nutrition from sources other than their plant hosts.

**Abstract:**

The glycoside hydrolase family 16 (GH16) is widely found in prokaryotes and eukaryotes, and hydrolyzes the β-1,3(4)-linkages in polysaccharides. Notably, the rice white tip nematode *Aphelenchoides besseyi* harbors a higher number of GH16s compared with other plant-parasitic nematodes. In this work, two GH16 genes, namely AbGH16-1 and AbGH16-2, were isolated and characterized from *A. besseyi*. The deduced amino acid sequences of AbGH16-1 and AbGH16-2 contained an N-terminal signal peptide and a fungal Lam16A glucanase domain. Phylogenetic analysis revealed that AbGH16-1 and AbGH16-2 clustered with ascomycete GH16s, suggesting AbGH16-1 and AbGH16-2 were acquired by horizontal gene transfer from fungi. In situ hybridization showed that both AbGH16-1 and AbGH16-2 were specifically expressed in the nematode gonads, correlating with qPCR analysis that showed the high transcript levels of the two genes in the female nematodes. AbGH16-1 and AbGH16-2 were also significantly induced in nematodes feeding on *Botrytis cinerea*. Characterization of the recombinant protein showed AbGH16-1 and AbGH16-2 displayed pronounced inhibition of both conidial germination and germ tube elongation of *B. cinerea*. In addition, silencing of AbGH16-1 and AbGH16-2 by RNA interference significantly decreased the reproduction ability of *A. besseyi* and had a profound impact on the development process of offspring in this nematode. These findings have firstly proved that GH16s may play important roles in *A.besseyi* feeding and reproduction on fungi, which thus provides novel insights into the function of GH16s in plant-parasitic nematodes.

## 1. Introduction

The rice white tip nematode *Aphelenchoides besseyi* is an ecto- or endoparasite that feeds on above-ground parts of host plants. This nematode has spread worldwide and caused extensive losses in global rice production. Symptoms caused by *A. besseyi* generally display the characteristic white tip of the top leaves, small grains, and erect panicles [1,2]. Damage caused by *A. besseyi* generally reduces rice yields by 12–20% with the annual economic loss estimated at $16 billion [3]. This nematode has also been found on a wide range of plants, including strawberry, tuberose, [4] and bird’s-nest fern [5], exhibiting a robust adaptivity to cope with various hosts.

In the field environment, *A. besseyi* can interact with a variety of plant pathogens, which consequently regulate plant health [6]. For instance, mixed infection by *A. besseyi* and *Curvularia lunata* largely deteriorates rice grain quality [7]. Frequent interactions inevitably provide an opportunity for *A. besseyi* to acquire other genes through horizontal gene transfer from fungi. In fact, *Aphelenchoides* spp. are also a kind of fungivorous nematode, feeding on several ascomycetes [8]. *A. besseyi* tends to multiply on the necrotrophic fungi served as a food source, such as *C. lunata* [9] and *Botrytis cinerea* [10]. In this scenario, *A. besseyi* may secrete a myriad of enzymes to degrade fungal cell walls to facilitate its feeding and reproduction [5].

In the past two decades, plant-cell-wall-degrading enzymes (CWDEs), such as cellulase [11,12], pectate lyase [13], polygalacturonase [14], and expansin [15,16], have been extensively identified in the sedentary plant root-parasitic nematodes. Likewise, the genes encoding fungal CWDEs have been discovered in several migratory fungus-feeding parasites, such as potato rot nematode *Ditylenchus destructor* [17], pine wood nematode *Bursaphelenchus xylophilus* [18,19,20], and *A. besseyi* [5,21]. One of the CWDEs, the glycoside hydrolase family 16 (GH16) is particularly abundant in *A. besseyi* based on a transcriptome analysis [22,23]. The GH16 family members can encode for β-1,3(4)-glucanases (EC 3.2.1.6), which hydrolyze the β-1,3(4)-linkages of polysaccharides and comprise a large and taxonomically diverse family of glycosidases and transglycosidases. Recently, nearly 23,000 GH16 proteins in CAZyme database were divided into 23 subfamilies with the sequence similarity network analysis [24]. However, to date only 2.5% of GH16 sequences have been enzymatically characterized, which challenges the functional prediction of GH16 proteins [25].

Growing evidence has shown the roles of GH16 are versatile. Industrial bacteria engineered with β-1,3(4)-glucanases promote polysaccharides degradation or biocontrol effects, exhibiting a favorable application foreground of GH16 [26,27]. GH16s in plants could be a susceptibility factor, because wheat plants with a silenced β-1,3(4)-glucanase gene reduced resistance to the aphid *Diuraphis noxia* [28]. Potential function of GH16 proteins is determined by the presence of catalytic residues and the site of expression [29]. Three GH16 proteins with β-1,3 glucanase activity were recently characterized in the European house dust mite *Dermatophagoides pteronyssinus*. β-1,3 glucanase activity was induced in *D. pteronyssinus* feeding on baker’s yeast. Also, the active β-1,3 glucanases were detected in the faeces of this mite, indicating GH16s in mites are capable of breaking down β-1,3 glucans of fungi [30]. GH16s in *Aedes aegypti* larvae also contain β-1,3-glucanase activity, and the expression levels of GH16s differed in larval head, gut, or rest of the body. The larvae with AeGH16.1 or AeGH16.6 knock-down by dsRNA soaking in vitro became highly sensitive to environmental stresses, while AeGH16.5 silenced larvae exhibited decreased activity in the gut, which then resulted in lower survival rates and pupation [31]. More recently, Li et al. identified a novel GH16 gene (Mbbg1) in the fungus-growing termite *Macrotermes barneyi*. Mbbgl encodes a endogenous digestive β-1,3(4)-glucanase and is mainly expressed in termite guts, suggesting its potential function in the decomposition of plant biomass and fungal hyphae [32].

Benefiting from the accumulating genome and transcriptome data, GH16s have been discovered in several nematode species, including root-parasitic nematode *Meloidogyne incognita*, *Pratylenchus coffeae*, the fungus-feeding nematode *A. besseyi* and *B. xylophilus* [23]. *B. xylophilus* harbors six predicted GH16 genes in its genome, and one GH16 (Bx-lam16A) was assumed to be acquired by horizontal gene transfer from bacteria [33]. The Bx-lam16A recombinant protein expressed in *Escherichia coli* preferentially hydrolyzed the β-1,3-glucan laminarin, suggesting their function in degrading β-1,3-glucan, one of the core components of the fungal cell wall [33]. Although there are a remarkable number of predicted GH16s in *A. besseyi*, to date few of these GH16s have been well characterized.

In the present study, we present the cloning and characterization of two GH16 genes (named AbGH16-1 and AbGH16-2) from *A. besseyi*. AbGH16-1 and AbGH16-2 are highly homologous with ascomycete fungal GH16 proteins. The expression of AbGH16-1 and AbGH16-2 was predominantly in the reproductive gland of females but they were also induced in the nematodes when feeding on *B. cinerea*. Recombinant proteins of AbGH16-1 and AbGH16-2 show inhibitory activity to *B. cinerea*. The silencing of AbGH16-1 and AbGH16-2 profoundly reduced nematode offspring, demonstrating that they are involved in nematode development and reproduction.

## 2. Materials and Methods

### 2.1. Nematode Preparation

The *A. besseyi* NJ isolate was initially obtained from infected rice plants in Jiangsu province of China, and subsequently cultured on *B. cinerea* grown on potato dextrose agar medium for three weeks at 25 °C [34]. The mix stages of nematodes were washed off and rinsed with deionized water containing penicillin (100 mg/mL), streptomycin (100 mg/mL), and amphotericin (0.25 mg/mL). The nematodes were cleaned by using 30% sucrose solution and then with water three times, followed by centrifuging the suspension at 3000 rpm.

### 2.2. AbGH16 Gene Discovery and Cloning

For gene discovery, we firstly constructed a transcriptome library of mix stages of *A. besseyi*. Total RNA was extracted from the active nematodes prepared as above using Trizol reagent (Invitrogen, San Diego, CA, USA) according to the manufacturer’s instructions. The quantity and quality of RNA samples were assessed using 1.2% agarose gel and examined with a Nanodrop 2000 spectrophotometer (Nanodrop, Wilmington, DE, USA). High quality total RNA (5 µg, 100 ng/μL) samples were sent to the Biomarker Biotechnology Corporation (Beijing, China) for RNA sequencing by Illumina HiSeq 2500. Cleaned RNA-Seq reads were mapped to the *B. xylophilus* Ka4 genome (https://parasite.wormbase.org/Bursaphelenchus_xylophilus_prjea64437/Info/Index/). Unique reads were aligned to a series of protein databases using blastx (E-value ≤ 10^−5^), including the NCBI non-redundant (Nr), the Swiss-Prot, the Trembl, the Kyoto Encyclopedia of Genes and Genomes (KEGG) and Gene ontology (GO) databases. The carbohydrate degrading enzymes, including glycoside hydrolase families (GH), were surveyed using dbCAN software with the parameters that e-values lower than 1e^−5^ were discarded. We then finally identified 18 GH families, wherein contained three GH16s (Table S1). Based on the partial sequence of the GH16s, gene-specific primers for AbGH16-1 were then designed by the Primer3 program (http://primer3.ut.ee/).

The first-strand cDNA was synthesized using HiscriptII 1^st^ Strand cDNA Synthesis Kit (Vazyme, Nanjing, China). The 3′ and 5′ terminal fragments were obtained by using the SMART™ RACE cDNA amplification kit (Clontech, Mountain View CA, USA). For 3′ end, GH16NJ-F1 and the universal primer (UPM) were used in a 25 μL reaction mixture, including 12.5 μL of 2× Taq Plus Master Mix (Vazyme, Nanjing, China), 2 μL of the GH16-F1/ UPM (10 mM each), and 0.25 μg cDNA template. Then 1 μL of the PCR product was used for a template, and the primers GH16NJ-F2/ NUP were used for the second amplification. For 5′ end, GH16NJ-R1/ UPM were used for the first round PCR temperate while GH16NJ-R2/ NUP for the second amplification. The PCR conditions were the same as those previously described. The entire sequence of AbGH16-1 was obtained by the primers GH16NJ-F.full and GH16NJ-R.full covering the ORF from the cDNA templates. AbGH16-2 cDNA was retrieved from NCBI with Genbank accession number KY290260, and the genomic DNA sequence of AbGH16-2 was produced using the primers GH16R1-21T.F/-21T.R. All the primers used in this study are listed in Table S2.

### 2.3. Sequence and Phylogenetic Analysis

The deduced protein sequence, PI (isoelectric point), and MW (molecular weight) of AbGH16-1/2 were calculated by EMBOSS Pepstats (https://www.ebi.ac.uk/Tools/seqstats/emboss_pepstats). The signal peptide of AbGH16-1/2 was determined using SignalP ver. 5.0 (http://www.cbs.dtu.dk/services/SignalP/) and motif analyses were performed using the Conserved Domain Database (https://www.ncbi.nlm.nih.gov/cdd/). Database searches of the nucleotide and deduced amino acid sequences were performed using the NCBI/GenBank/Blast. Multiple sequences were aligned by MAFFT (https://www.ebi.ac.uk/Tools/msa/mafft/), and the output file was modified using BoxShade (https://embnet.vital-it.ch/software/BOX_form.html).

The phylogenetic relationship of AbGH16-1/2 protein with other GH16s was revealed using IQ-TREE web server (W-IQ-TREE) by the Maximum Likelihood approach [35]. A total of ultrafast bootstrap (1000 replications) were used to test topology. The models for phylogenetic analysis were automatically selected by IQ-TREE program. The visualization and modification of phylogenetic trees were performed using the FigTree 1.4. Ultrafast bootstrap values (>50) were reported with each branch, and the taxonomic origins of the sequences were shown in different colors.

### 2.4. In Situ Hybridization

The fragments used as probes were separately amplified from the full-length cDNA of *A. besseyi* by the specific primer pair GH16NJ-F3/R3 (for AbGH16-1) and GH16R1-F/R (for AbGH16-2). The digoxigenin (DIG)-labelled sense and antisense probes were synthesized by the asymmetric PCR with the PCR DIG Probe Synthesis kit (Roche Diagnostics GmbH, Mannheim, Germany). In situ hybridizations were conducted as described earlier [36] with minor modification. About fifty thousand mixed stages of *A. besseyi* were fixed in 4% paraformaldehyde at 4 °C for 16 h, followed by 4 h at room temperature and cut into 2–5 pieces. The hybridization was performed at 45 °C overnight. Sense probes were used systematically to control the specificity of the hybridizations.

### 2.5. Differential Expression in Developmental Stages and Feeding Progress

To survey mRNA levels at different growth stages of the nematode, eggs of *A. besseyi* were collected using the method described by Yoshida et al. [10]. The eggs were surface sterilized using a 10% bleach solution, and then incubated in sterilized tap water at room temperature for 48 h. A large number of hatched second-stage juveniles (J2) were collected and transferred to the *B. cinerea* hyphae mat cultured on PDA medium. The third-stage J3, fourth-stage J4, and adults were collected using a Baermann funnel after 1 d, 3 d, and 7 d, respectively. Male and female nematodes were subsequently separated from the adults using a dissecting needle under a microscope. To survey the expression of GH16 genes in response to feeding progress, 200 μL ddH2O containing 1000 active mixed stages of nematodes (juvenile: male: female = 20%: 20%: 60%) were added on the white hyphal mat of *B. cinerea*. Each Petri dish was placed with three batches of nematodes, which were then collected by washing with ddH2O after 0, 1, 3, 6, 12, 18, and 24 hours separately.

Total RNA of the nematodes was prepared by using the Trizol reagent. First-strand cDNAs were synthesized using PrimeScript™ RT reagent kit with gDNA Eraser, according to the manual (Takara Bio, Beijing, China). Quantitative real-time PCR (qPCR) was then conducted in triplicates with a LightCyler^®^96 (Roche Applied Science, Germany) system using the SYBR^®^ Premix Ex Taq™ II (Tli RNaseH Plus) (Takara Bio, Beijing, China). The specific primer pairs of qGH16NJ-F/R and qGH16R1-F/R were used to monitor the transcription abundance of AbGH16-1 and AbGH16-2 genes separately, while the 18S rDNA of *A. besseyi* amplified by the primers qAb18S-F and qAb18S-R served as the reference gene. The relative quantity of gene expression was detected using 2^−ΔΔCT^ method.

### 2.6. Heterologous Expression of AbGH16 Proteins

The AbGH16-1 and AbGH16-2 coding sequence without the predicted signal peptide-encoding region were cloned into expression vector pET32T and pGS21T (Probegene, Xuzhou, China), respectively. The resulting plasmids were validated by the direct sequencing of PCR products and then transformed into an *Escherichia coli* Rossetta (DE3) competent cell. Isopropyl β-D-1-thiogalactopyranoside (IPTG) was added at final concentration of 0.5 mM and then incubated at 15 °C for 15 hrs. The bacterial cells were pelleted by centrifugation and the protein production was analyzed on 12% SDS-PAGE. To purify the recombinant proteins, bacterial culture from each treatment was pelleted by centrifugation at 4000 g for 15 min. Then the pellets were dissolved in the lysis buffer (20 mM Tris-HCl, 50 mM NaCl, 0.1% Triton-100, pH 8.0), and sonicated (600–800 W, 30 min) to break up the bacteria. After centrifugation at 4 °C, 12,000 rpm for 20 min, the supernatant of the sonicated fraction was washed and eluted with the Ni-NIC column (Probegene, Xuzhou, China). The concentration of the purified proteins was measured by using a BCA protein assay kit.

### 2.7. Antifungal Activity Assays

Fungal spores were harvested with sterilized water from PDA plates containing *B. cinerea* cultures. The spore suspension was centrifuged at low speed and then adjusted to a concentration of 3 × 10^5^ spores in 1 mL PYD. The AbGH16-1 and AbGH16-2 solutions were separately added into the spore suspension in a sterile Eppendorf tube with the final protein concentration at 10 μg/mL and incubated at 25 °C for various periods (6 h to 12 h) before microscopic examination. The protein solution was replaced with an equal volume of sterile distilled water as the control. The conidial germination and germ tube growth were observed and measured on a microscope slide. The values obtained for the control (no enzyme) were taken as 0% inhibition, and all other values were divided by these values and multiplied by 100 to obtain percent inhibition.

### 2.8. RNA Interference

In vitro transcriptions were performed using a T7 RNAi Transcription Kit (Vazyme, Nanjing, China) according to the manufacturer’s instructions. The two primer pairs (GH16- T7.F3/ R3 and GH16-F3/ T7.R3; GH16R1- T7.F /R and GH16R1-F/T7.R) were designed to amplify the sense and antisense single-stranded RNA (ssRNA) derived from the cDNA separately. The PCR products were mixed in equal proportion, incubated at 37 °C for 3 h. After enzyme digestion with DNase I and RNase T1, the dsRNA was then subjected to quantity and quality evaluation by Nanodrop 2000 and on 2.0% agarose gel electrophoresis. A segment of non-endogenous green fluorescent protein (GFP) (500 bp) was amplified with the specific primers to serve as a negative control.

For RNAi, 1000 active nematodes were washed three times using RNase-free water, and were then soaked in 50 μL of the soaking buffer (0.25 × M9, 1% resorcinol, 2 μg/μL dsRNA) and incubated in the dark at 25 °C for 24 h. Nematodes soaked in the GFP-derived dsRNA solution and only in the soaking buffer without dsRNA served as controls. Nematodes were then washed five times with ddH_2_O to remove the components of soaking buffer. The efficiency of GH16 knockdown was verified by qPCR as described previously. To test the feeding and reproductive capacity of GH16-silenced nematode, 500 treated nematodes were cultured on *B. cinerea* grown on PDA for 15 d. The nematodes were harvested by washing them down from the Petri dishes and were then counted under a stereo microscope.

## 3. Results

### 3.1. Identification of the Novel GH16 Gene in the A. besseyi

**Figure 1:**
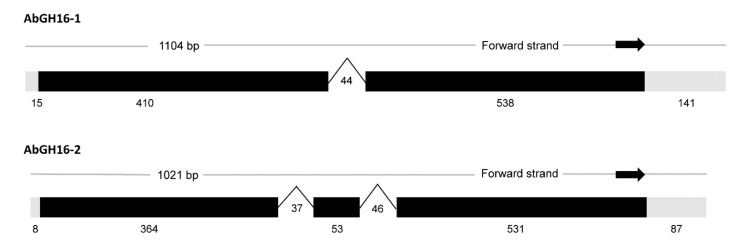
The gene structures of two GH16 genes in *A. besseyi.* One 44 bp-sized intron was found in AbGH16-1, while two introns with 37 bp and 46 bp separately, were present in the AbGH16-2.

A 1104 bp putative GH16 mRNA sequence was assembled by inverse and RACE-PCR and ended with a 31 poly-A tail from the *A. besseyi* NJ isolate, which we designated AbGH16-1 (Genbank number: MF326215). The ORF start codon was predicted by the ORF finder and consisted of a 945 bp ORF sequence before the stop codon. The theoretical PI and MW of AbGH16-1 protein, predicted by ProtParam, were 6.90 and 35.37 kDa respectively. A signal sequence cleavage site was between residues 18 and 19. Another GH16 gene (Genebank number: KY290260), designed as AbGH16-2, from *A. besseyi* R1 isolate contained 1019 bp length sequences with a 945 bp ORF. AbGH16-2 protein also had a signal peptide (1–18 aa), and its predicted PI and MW were 6.99 and 35.52 kD respectively. A genomic clone of AbGH16-1 and AbGH16-2 were obtained by PCR amplification from *A. besseyi* gDNA. The exon/intron boundaries of the genomic sequences were determined by aligning the genomic sequence with the corresponding cDNA sequences. One intron was found in AbGH16-1 genomic DNA, two introns in AbGH16-2 (Figure 1).

**Figure 2:**
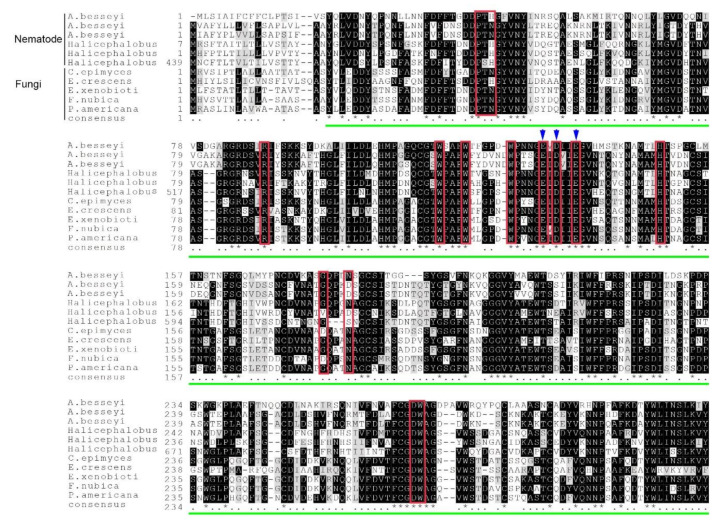
Multiple sequence alignment of the predicted AbGH16 protein with other GH16 proteins. Six nematode GH16 proteins in *Aphelenchoides besseyi* isolates NJ (AbGH16-1; AWH98111), R1 (AbGH16-2, ARD05880), and Fm (ARD05881), and in *Halicephalobus* sp. (KAE9549822, KAE9555931, KAE9549822, and five GH16s in the fungal species including *Capronia epimyces* (XP_016224388), *Emmonsia crescens* (PGH36908), *Exophiala xenobiotica* (XP_013314421), *Fonsecaea nubica* (XP_022496328), *Phialophora americana* (KIW68755) were aligned by MAFFT. The red box indicates catalytic residues, the blue arrow refers to EXDXXE active sites of GH16 protein, and the green line indicates the LamG domain.

A BlastP search showed that the novel AbGH16-1 protein (AWH98111) was similar to two known β-1,3(4)-glucanase of *A. besseyi* (ARD05880, and ARD05881) with 49.52% sequence identity. However, a higher similarity was found in the free-living nematode *Halicephalobus* sp. (KAE9549822, 52.87%; KAE9555931, 50.32%; KAE9549822, 49.84%) and in several fungi, especially to the environmental and plant ascomycetes, such as *Exophiala xenobiotica* (XP_013314421, 54.49%), *Fonsecaea nubica* (XP_022496328, 54.36%), *Phialophora americana* (KIW68755, 53.95%), *Capronia epimyces* (XP_016224388, 53.16%), and *Emmonsia crescens* (PGH36908, 52.38%). Multiple sequence alignment showed the consensus features of GH16 proteins containing the catalytic residues with EXDXXE active sites, and a LamG superfamily domain, which is specific to GH16 fungal Lam16A glucanase (Figure 2).

### 3.2. Phylogenetic and Structural Analysis

**Figure 3:**
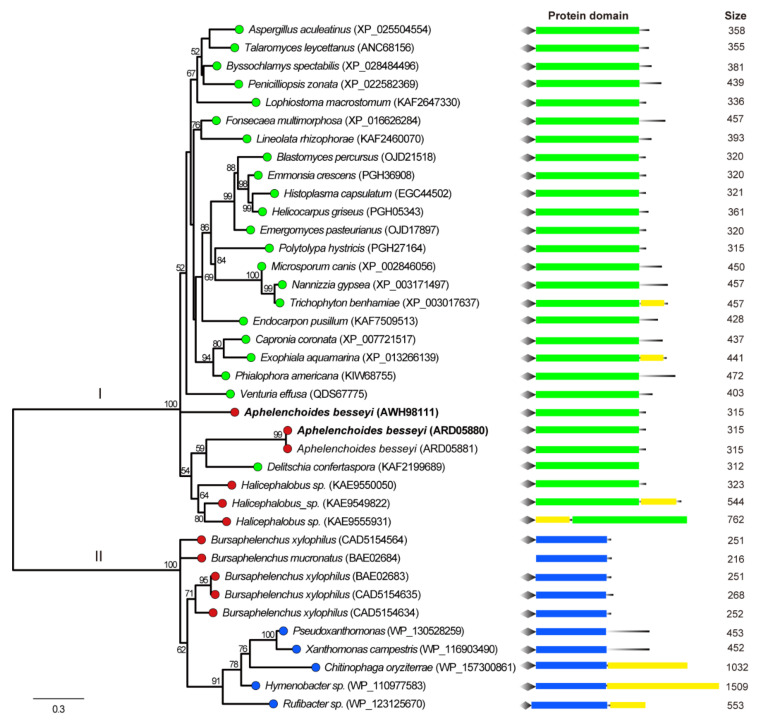
Phylogenetic relationship of nematode GH16 proteins and their relatives from the NCBI database. The branch length of the maximum likelihood tree indicates evolutionary distance. One thousand bootstrap replicates were performed, and the node labels represent the percentage of bootstrap support. The branches with different colored circles correspond to different taxonomic groupings of the species: red = nematodes, green = fungi, and blue = bacteria. *A. besseyi* GH16-1 and AbGH16-2 is highlighted in bold. The conserved regions and the relative positions of domians in the GH16 protein are shown. Diamond = signal peptide, green rectangle = fungal-specific Lam16A glucanase domain, blue rectangle = laminarinase-like domain, yellow rectangle = other domain(s). The black line at the end of the protein domain schema indicates the relative position of the domains with respect to the C-terminal of the protein.

A Maximum Likelihood phylogenetic tree was constructed using GH16 proteins from the selected hits, known GH16 proteins in *B. xylophilus*, and their similar sequences from NCBI and CAZy database. The phylogenetic analysis showed that three available AbGH16 proteins and three predicted GH16s of *Halicephalobus* sp in the database were grouped into the fungi origin (Clade I), while *B. xylophilus* GH16 and *B. mucronatus* GH16 were in bacteria origin group (Clade II) (Figure 3). In each group, the phylogenetic topology was not congruent with that of a species tree for these species, suggesting that GH16s in plant nematodes were from ascomycete fungi. In addition, protein domain structure was investigated by scanning against the CDD database, and the result showed that each GH16 member in Clade I herein has a Lam16A glucanase domain, while the members in Clade II have a laminarinase-like domain (Figure 3).

### 3.3. In Situ Hybridization and Expression Analysis

**Figure 4:**
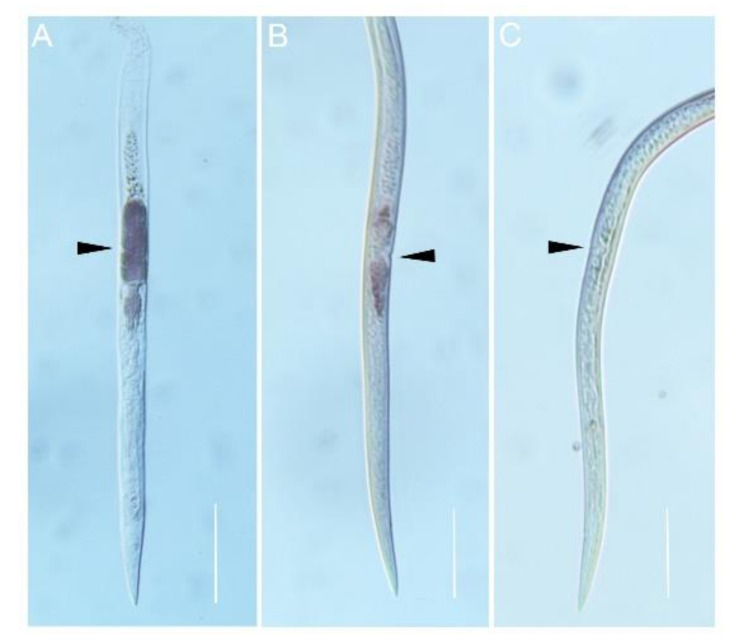
Localization of AbGH16 gene expression in *A. besseyi* using in situ hybridization. AbGH16-1 (**A**) and AbGH16-2 (**B**) probes were extremely strong with specific dark staining in the female gonads, while no such staining pattern was observed in the negative control (**C**). The black arrows indicate female gonads. Scale bar = 50 μm.

**Figure 5:**
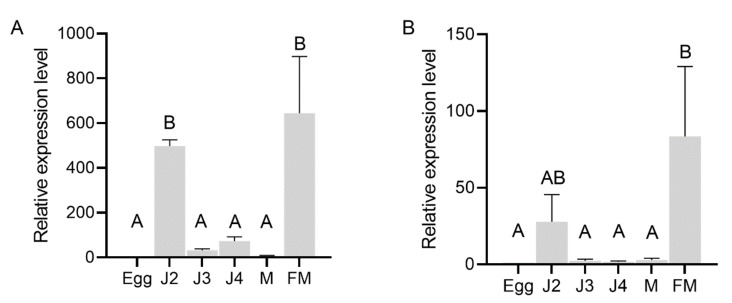
Relative expression level of AbGH16s in different developmental stages of *A. besseyi*. (**A**) AbGH16-1; (**B**) AbGH16-2. The bars indicate the standard deviation of mean (*n* = 3) and different letters on the bar indicate significant differences (*p* < 0.01) between nematode specimens by Tukey’s test. M = male and FM = female.

In situ hybridization was performed to localize the expression of AbGH16-1 and AbGH16-2 in the mix developmental stages of *A. besseyi*. The DIG-labelled antisense cDNA probe of AbGH16-1/2 specifically hybridized with the mRNA accumulated in the adult female gonad cells. No hybridization signal was detected when the control sense probes were used (Figure 4). To further investigate the expression pattern of AbGH16-1 and AbGH16-2 during different developmental stages of *A. besseyi*, we used cDNA generated from nematode RNA isolated at developmental stages (eggs, J2, J3s, J4s, males and females) in qPCR analyses. The qPCR analysis showed that transcription levels of AbGH16-1 and AbGH16-2 varied with the development stage of *A. besseyi*. AbGH16-1 and AbGH16-2 was highly expressed in J2 and females, but poorly expressed in the eggs, J3, J4, and males (Figure 5).

### 3.4. GH16 Expression was Induced in A. besseyi Feeding on the Fungus

**Figure 6:**
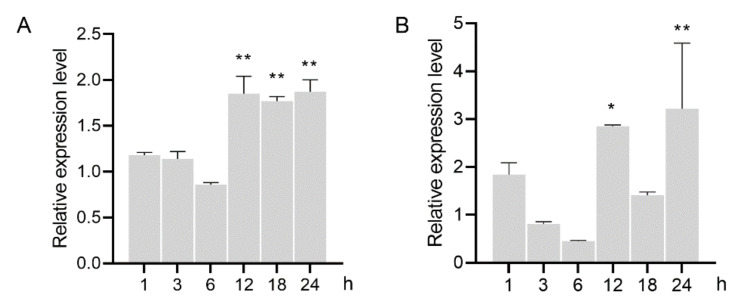
Relative mRNA expression levels of GH16s in *A. besseyi* feeding on *Botrytis cinerea*. (**A**) AbGH16-1; (**B**) AbGH16-2. The bars indicate the standard deviation of mean (*n* = 3), asterisks on the bar indicate significant differences (*, *p* < 0.05; **, *p* < 0.01) between 0 h and the corresponding time point by Student’s *t*-tests.

To determine the role of GH16 in the process of nematode feeding, the relative expression level was analyzed in the nematodes inoculated on hyphal mat of *B. cinerea*. The results showed that expression of both AbGH16-1 and AbGH16-2 were slightly upregulated in the nematodes 1 h after feeding (HAF), and a downregulate expression was found 3 to 6 HAF. The transcripts of AbGH16-1 were accumulated from 12 to 24 HAF (*p* < 0.01), while AbGH16-2 was also highly induced 12 and 24 HAF, but not significant expressed 18 HAF (Figure 6).

### 3.5. In Vitro Antifungal Activity of Purified Recombinant AbGH16s

**Figure 7:**
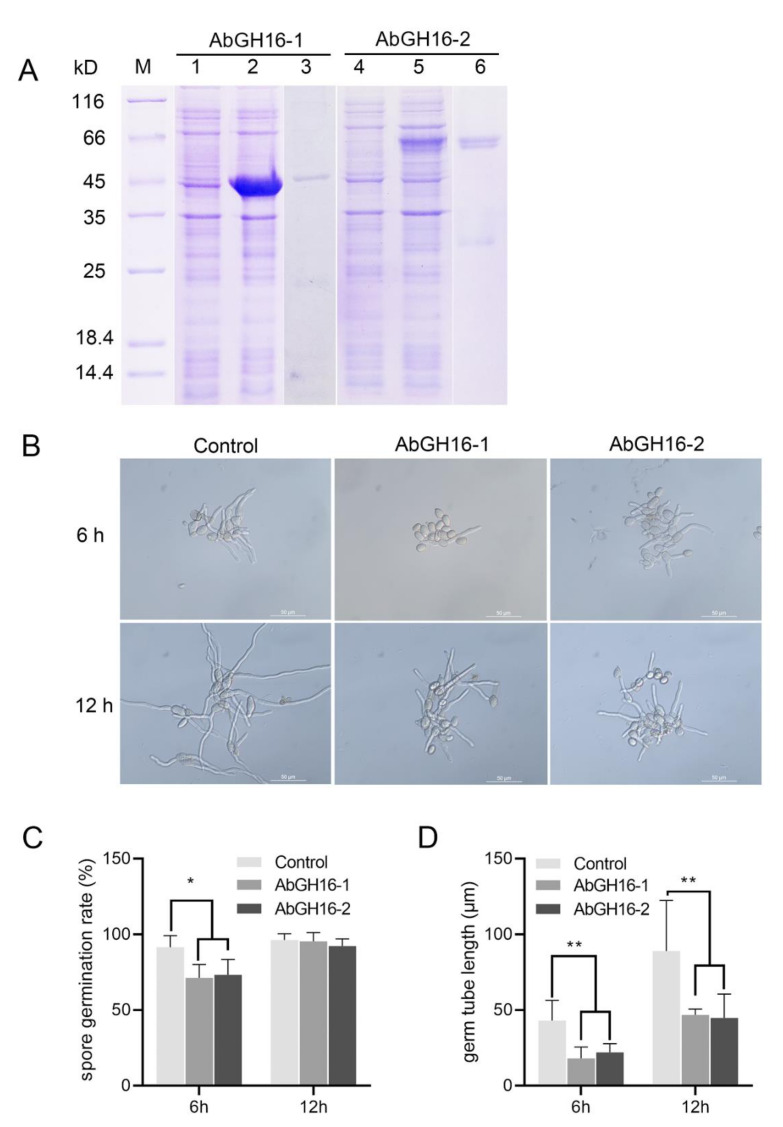
Antifungal activity assay of the AbGH16 recombinant protein. (**A**): The 12% SDS-PAGE analysis of AbGH16 expression in Escherichia coli. Lane M, molecular mass marker; Lane 1, 4, lysate of the non-induced *E. coli* cells expressing AbGH16-1 and 2; Lane 2, 5, lysate of the IPTG-induced *E. coli* cells expressing AbGH16-1and 2; Lane 3, 6, purified proteins of AbGH16-1 and AbGH16-2. (**B**): Germ tube elongation of *Botrytis cinerea* treated by AbGH16 enzymes. 10 μg purified protein of AbGH16-1 and AbGH16-2 were mixed separately with 1 ml of a *B. cinerea* spore suspension (3 × 10^5^ spores) in YEPD medium, and incubated at 25 °C for 6 h and 12 h; the same concentration of spore suspension without addition of the proteins was used for a control (3); scale bar = 50 µm. C-D: inhibition of the spore germination rate (**C**) and germ tube elongation (**D**) of *B. cinerea*. The data from two experiments with four replicates per treatment were pooled. Asterisks on the bar indicate significant differences (*, *p* < 0.05; **, *p* < 0.01) between treatments as determined by Student’s *t*-tests.

To determine the antifungal activity, the recombinant proteins AbGH16-1 and AbGH16-2 were produced using *E. coli* cells. The mass of the recombination protein AbGH16-1 with TRX tag and -2 with GST tag were about 47 kD and 60 kD separately (Figure 7A). The biological activity of the purified enzymes was then tested for activity against the conidial germination and of germ tubes elongation of *B. cinerea*. Most of the number of conidia (91.53%) in control (no enzyme) germinated 6 h after inoculation, while AbGH16-1 and AbGH16-2 treatments separately resulted in 20.25% and 18.19% inhibition of conidia germination. Thus, the elongation of germ tubes was significantly restrained, in contrast to the control (Figure 7B,C,D). Spore germination rates were no longer disturbed after 12 h treatment with the enzymes. However, the length of germ tubes continued to be significantly suppressed (Figure 7B,C,D)

### 3.6. Decreased Reproduction Ability in the GH16 Knockdown Nematodes

**Figure 8:**
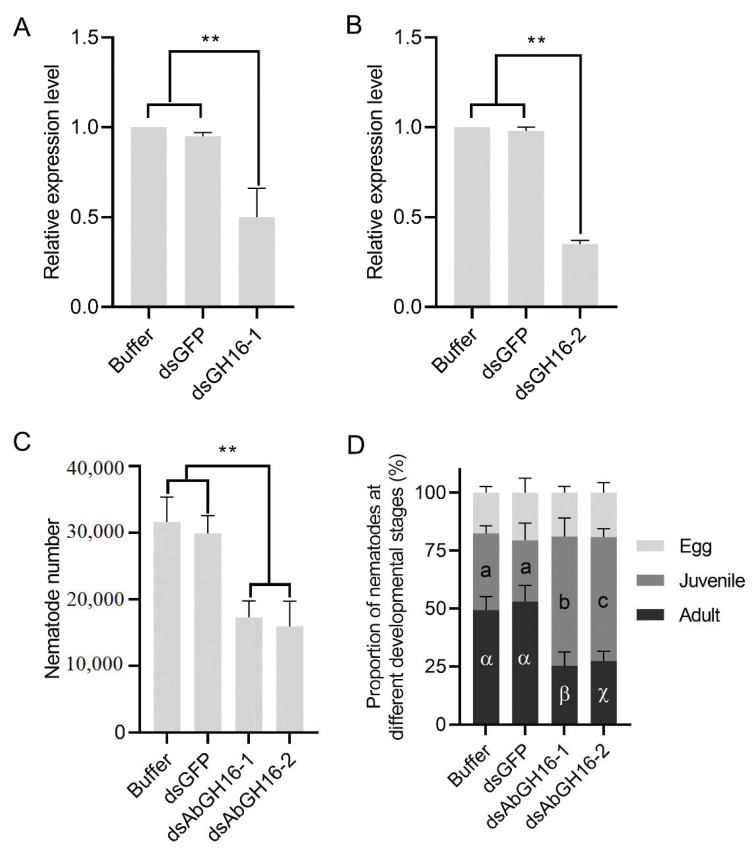
Effects of RNAi in vitro on nematode development and reproduction. A, B, Relative expression levels of AbGH16-1 (**A**) and AbGH16-2 (**B**) under different treatments. (**C**), Total numbers of *A. besseyi* cultured on *B. cinerea* for 15 d. (**D**), proportion of the nematodes in different developmental stages. Asterisks (**) indicate significant differences based on Student’s *t* test. (*p* < 0.01). The Latin and Greek letters separately indicate significant differences between treatments based on Tukey’s test (*p* < 0.05). Error bars indicate standard deviation.

In vitro RNAi targeting of AbGH16-1 AbGH16-2 was performed to analyze whether AbGH16-1 or AbGH16-2 plays an important role in nematode growth and reproduction. RNAi efficiency was firstly evaluated by means of qPCR. AbGH16-1 and AbGH16-2 were dramatically reduced when exposed to AbGH16-1/2 derived dsRNA solution for 24 h, suggesting that the transcription of AbGH16-1/2 had been effectively depleted (Figure 8A,B). The nematodes with AbGH16-1/2 knock-down thus produced significantly less offspring, compared to the nematodes treated with buffer or GFP dsRNA (Figure 8C). In addition, treatment of *A. besseyi* with AbGH16-1/2 dsRNA significantly reduced the proportion of adults, and increased the proportion of juveniles but had no effects on the proportion of eggs (Figure 8D).

## 4. Discussion

GH16 members have been found across all domains of life, including bacteria, oomycetes, fungi, plants, and animals (insects and other invertebrates) [24]. However, GH16 seems exist specifically in certain nematode species. Recent comparative transcriptomic analysis indicated that GH16 members are in *A. besseyi*, *A. ritzemabosi*, *B. xylophilus*, *Radopholus similis*, *P. coffeae*, and *M. incognita* but not in *Globodera pallida*; wherein, *A. besseyi* has an abundance of GH16s (90 contigs), and *B. xylophilus* has only 6 contigs [23]. The presence of the considerable number of GH16 family sequences in *A. besseyi* suggests that this gene family has versatile functions during nematode growth and reproduction.

In the present study we described the cloning and characterization of two GH16 genes, namely AbGH16-1 and AbGH16-2 separately, from the rice white tip nematode *A. besseyi.* The presence of polyA tail at the 3′end of AbGH16-1 and AbGH16-2 cDNA, the presence of introns within the corresponding AbGH16-1 and AbGH16-2 genomic DNA fragments and the predicted signal peptides in AbGH16-1 and AbGH16-2 protein indicated that AbGH16-1 and AbGH16-2 originates from eukaryotes excluding the possibility of bacterial contamination from a close association with *A. besseyi*. In general, the GH16 proteins from fungi share the conserved amino acid motif EXDXXE, while bacteria share the EXDXE motif [37]. Both AbGH16-1 and AbGH16-2 encode β-1,3(4)-glucanases that have a fungal Lam16A glucanase domain with the EXDXXE active-site (Figure 2 and Figure 3). Therefore, the characterized AbGH16-1 and AbGH16-2 are likely from fungi and are implicated in degrading the fungal cell wall. In addition, three GH16s with fungal Lam16A glucanase domains were also found in the draft genome of *Halicephalobus* (strain NKZ332), a parthenogenic nematode isolated from termites [38]. Because *Halicephalobus* sp. can specifically colonize rotting wood, the existence of GH16s suggest this nematode may employ a complicated strategy to degrade fungal cell walls.

A phylogenetic tree was constructed to further understand the evolutionary history of GH16s. Although the facultative fungal feeders *A. besseyi*, *B. xylophilus,* and *B. mucronatus* share the order Aphelenchida and have the closest genetic relationship, as evident from their small subunit ribosomal DNA (SSU rDNA) sequences, all the known GH16s from *A. besseyi* share the same clade I with ascomycetes, independent of the clade II of *B. xylophilus* and *B. mucronatus* and bacterial species located (Figure 3). To date GH16 has comprised 27 subfamilies as defined in CAZy database, wherein AbGH16-1 and AbGH16-2 are classified as GH16 subfamily 1, while BxGH16 (BAE02683) from *B. xylophilus* and BmGH16 (BAE02684) from *B. mucronatus* are grouped into the subfamily 3 [24]. Interestingly, unlike GH16 subfamily 3, the subfamily 1 comprises exclusively fungal enzymes except for a few members from *A. besseyi* [24]. Previous studies have shown that a GH16 (BxLAM16A) from *B. xylophilus* is similar to bacterial sequences, and hypothetically originated from bacteria that were closely associated with the ancestors of *B. xylophilus* [33]. Given that GH classified into the same subfamily are generally thought to have evolved from a common ancestor [39], the fungivorous nematode *A. besseyi* probably acquired AbGH16-1 and AbGH16-2 genes via lateral gene transfer from feeding on fungi.

In situ hybridization signals of AbGH16-1 and AbGH16-2 were specifically highlighted on the reproductive tracts in the spermatheca of *A. besseyi* adult female, unlike BxLAM16A, which solely expressed in the esophageal gland cells of female *B. xylophilus* [33]. It’s not surprising, however, several GH members have been observed in reproductive systems of plant nematodes, such as GH5 from *A. besseyi* [5], and GH18 from *B. xylophilus* [19]. The GH5 cellulase gene AbeGH5-1 is localized in the ovary and testis of *A. besseyi*, while the GH18 chitinases gene *Bx-chi-1* is similarly expressed in the spermatheca of *B. xylophilus*, indicating their role in nematode reproduction. As the spermatheca is the reproductive organ where spermatozoa are transferred into and stored for fertilization, therefore, AbGH16-1 and AbGH16-2 are also presumed to be associated with the nematode reproduction process.

The expression analysis in different developmental stages of *A. besseyi* found that AbGH16-1 and AbGH16-2 were highly expressed in females, but had low transcript levels in eggs, juveniles (J3–J4), and males, which were in line with the results of in situ hybridization. Notably, expression level of AbGH16-1 and AbGH16-2 also largely increased in J2, although in situ hybridization signals were not detected in any cells of J2. Danchin et al. [40] found the GH proteins with CBM2 module were not only expressed in the migratory J2 of root knot nematodes, but were also induced in the vulva region of the sedentary females. Generally, J2 was recognized as the initial infective stage of plant nematodes. In the early stage of foraging, J2 may secrete cell-wall-degrading enzymes to facilitate feeding. In this study, J2 was hatched from the mat of *B. cinerea*, and then J2 easily obtained the fungal food source. Like other cell-wall-digesting enzymes related to infection and parasitism of plant nematodes, such as pectate lyases in cyst and root-knot nematodes [41], expansin-like protein in *Heterodera avenae* [16] and GH12 cellulase in *Xiphinema index* [42], the transcripts of AbGH16-1 and AbGH16-2 were highly induced in the mixed stages of *A. besseyi* after 12 h feeding on *B. cinerea* (Figure 6). We therefore speculate AbGH16-1 and AbGH16-2 might be briefly induced in J2. Although more evidence is needed, AbGH16-1 and AbGH16-2 at least partially exhibited the potential role in the feeding of *A. besseyi*, which may promote egg production in the female nematodes.

Given the essential role of GH16 in degrading cell wall, the active recombinant AbGH16-1 and AbGH16-2 was obtained by induction at optimal conditions. Intriguingly, the purified AbGH16-1 and AbGH16-2 showed potent inhibition effects on conidial germination and germ tube elongation (Figure 7). The fungal cell is encased within a complex matrix of interconnected polysaccharides and proteins, which constitute the fungal cell wall [43]. Evidence showed that GH16s from different fungi and bacteria can degrade various polysaccharides. A previous study indicated that expression of *Paenibacillus polymyxa* β-1,3(4)-glucanase in *Streptomyces lydicus* improves its biocontrol effect against *B. cinerea* [27]. Jijakli et al. [44] found the activity of GH16 (exo-β-1, 3-glucanase) produced by *Pichia anomala* largely upgraded the media containing a cell wall preparation of *B. cinerea* as the sole carbon source, suggesting that GH16 activity is one of the mechanisms of action involved in the suppression of *B. cinerea*. Therefore, it is probable that AbGH16-1 and AbGH16-2 cause the degradation of the essential polysaccharides of fungal cell walls, which further resulted in the abnormal growth of *B. cinerea*.

Although hallmarks of cell wall modification proteins have been studied extensively in nematodes, to the best of our knowledge, characterization of GH16s using reverse genetic tools has received limited attention. Recently, Souza et al. [31] identified six GH16 genes from *A. aegypti* larvae and found knock-down of AeGH16.5 reduce the activity of β -1,3-glucanase, which led to significantly lower survival and pupation rates than the controls. RNA interference assays on transcripts encoding AbGH16-1 and AbGH16-2 have no impact on nematode survival. However, the nematodes with AbGH16-1/2- knocked down when culturing on *B. cinerea* produced less offspring. Notably, proportions of the juveniles and adults (males and females) were markedly reduced in dsGH16- 1/2 treated nematodes, compared to the soaking buffer or dsGFP treated control. This could be explained by the RNAi-mediated knockdown of AbGH16-1/2 have profound impacts on the offspring, which impeded the developmental process from eggs to adults in *A. besseyi*.

## 5. Conclusions

Both AbGH16-1 and AbGH16-2 have a fungal Lam16A glucanase domain, and presumably originate from fungi by means of a horizontal gene transfer. The combined results from the gene discovery, localization, expression, recombinant protein characterization, and silencing, suggesting that AbGH16-1 and AbGH16-2 may play key roles in nematode reproduction, and are at least partially involved in the early feeding of *A. besseyi*. These findings have proved for the first time that GH16s are required for feeding and reproduction in plant parasitic nematodes. In consideration of the remarkable abundance in *A. besseyi* and its destructive role as a plant pathogen, more nematode GH16 members need to be discovered and further characterized.

## Data Availability

The data presented in this study are available on request from the corresponding author.

## Supplementary Materials

Supplementary materials can be found at https://www.mdpi.com/2076-2615/11/2/374/s1, Table S1: GH genes survey in *Aphelenchoides besseyi* transcriptome data, Table S2: Primers used in this study.
