# Supplementary material for: Characterization and Functional Importance of Two Glycoside Hydrolase Family 16 Genes from the Rice White Tip Nematode Aphelenchoides besseyi
Source: Animals (Basel). 2021 Feb 2;11(2):374. doi: 10.3390/ani11020374 (PMC7913077; doi:10.3390/ani11020374)
Supplement: Supplementary file 1 [file animals-11-00374-s001.pdf]

animals

MDPI

Supplementary Materials: Characterization and Functional Importance of Two Glycoside Hydrolase Family 16 Genes from the Rice White Tip Nematode *Aphelenchoides besseyi*

Hui Feng, Dongmei Zhou, Paul Daly, Xiaoyu Wang and Lihui Wei

Citation: Feng, H.; Zhou, D.; Daly, P.; Wang, X.; Wei, L. Characterization and functional importance of two glycoside hydrolase family 16 genes from the rice white tip nematode *Aphelenchoides besseyi*. *Animals* **2021**, *11*, 374. https://doi.org/10.3390/ani11020374

Academic Editor: Giada D'Errico, Maria Paola Maurelli, Francesco Vinale

Received: 7 December 2020
Accepted: 31 January 2021
Published: 2 February 2021

Publisher's Note: MDPI stays neutral with regard to jurisdictional claims in published maps and institutional affiliations.

Copyright: © 2021 by the authors. Licensee MDPI, Basel, Switzerland. This article is an open access article distributed under the terms and conditions of the Creative Commons Attribution (CC BY) license (http://creativecommons.org/licenses/by/4.0/).

Table S1. GH genes survey in *Aphelenchoides besseyi* transcriptome data.

GHs	Transcript ID	E-value
GH2	trans117704_10_47-1081	1.00×10^{-45}
GH2	trans679_10_1-1425	7.20×10^{-64}
GH13	trans794_19_3-1889	2.20×10^{-128}
GH16	trans152380_4_1-207	2.50×10^{-06}
GH16	trans32403_1_2-244	2.30×10^{-08}
GH16	trans7912_10_102-1073	3.60×10^{-20}
GH18	trans168_7_148-990	4.40×10^{-37}
GH19	trans1382_5_3-800	8.10×10^{-42}
GH20	trans113061_12_3-1928	1.40×10^{-28}
GH20	trans1978_11_210-1763	4.10×10^{-22}
GH20	trans3367_16_93-1748	6.60×10^{-81}
GH29	trans3842_11_21-1463	1.70×10^{-96}
GH30	trans117090_10_1-1596	4.10×10^{-152}
GH30	trans125092_10_3-1589	1.50×10^{-148}
GH31	trans4754_18_1-2748	9.50×10^{-152}
GH35	trans114947_13_86-2071	2.20×10^{-103}
GH35	trans124383_7_2-568	8.00×10^{-46}
GH37	trans120565_17_3-1877	4.00×10^{-162}
GH38	trans116548_13_4-1611	7.00×10^{-57}
GH38	trans8134_31_573-3653	3.10×10^{-59}
GH45	trans123487_5_2-538	3.90×10^{-76}
GH47	trans113602_22_314-2146	9.40×10^{-150}
GH47	trans3198_12_16-1710	6.20×10^{-147}
GH47	trans3658_22_101-3109	1.90×10^{-138}
GH56	trans122501_7_2-805	2.60×10^{-66}
GH85	trans7967_9_5-1405	9.90×10^{-67}
GH99	trans115030_16_242-1630	4.10×10^{-120}
GH109	trans114061_12_444-1505	2.50×10^{-7}
GH109	trans119448_8_62-1099	4.10×10^{-12}
GH109	trans2441_5_2-700	9.40×10^{-12}

Table S2. Primers used in this study.

Primer ID	Primer Sequence (5′–3′)	Aim For
GH16NJ-F1	CGACGATGCTTTCTATTGCT	RACE-PCR for AbGH16-1
GH16NJ-R1	CAATAACAACACAGTCTCTC	
GH16NJ-F2	GGACAATGCGGAACTTGGAGTGC	
GH16NJ-R2	CGCCATGTAGACACCACCCTTCT	
UPM	CTAATACGACTCACTATAGGG-CAAGCAGTGGTATCAAC-GCAGAGT (long); CTAATAC-GACTCACTATAGGGC (short)	
NUP	AAGCAGTGGT AACAACGCAGAGT	
GH16NJ-F.full	ACATGGGGACGACGATGCT	Full length of AbGH16-1
GH16NJ-R.full	CACTTGCTTGAATTGATTTA	
qGH16NJ-F	ACAGTGCGATTTGAATGCCA	qPCR for AbGH16-1
qGH16NJ-R	TTGAGGATATTGCCGCCAGA	
qAb18S-F	TGCGGTAATTCTGGAGCCAA	qPCR (reference gene)
qAb18S-R	CCCGTTAGAACCATGGCAGT	
GH16NJ-21T.F	CATGCCATGGCTTATCAGTT-GGTCGACAACTATCA	Plasmids construction for protein expression
GH16NJ-21T.R	CATGCTCGAGTTATTTCTTATA-GATTTTGAGTGAATTGATCAAC-CAA	
GH16R1-21T.F	CATGCCATGGCTTATCGCTT-GGTCGACAATTACACA	
GH16R1-21T.R	CATGCTCGAGTTAGTTTTGA-TAAACTTTCAGCGAATTGA	
GH16NJ-F3	GGACAATGCGGAACTTGGAG	ISH/ RNAi for AbGH16-1
GH16NJ-R3	AGCTGCCAGACATTGAGGAT	
GH16NJ-T7.F3	T7 adapter- GGACAATGCGGAACTT-GGAG	RNAi for AbGH16-1
GH16NJ-T7.R3	T7 adapter- AGCTGCCAGACATT-GAGGAT	
GH16R1-F	GCGAATCCAAATCACAGGCA	ISH/qPCR/RNAi for AbGH16-2
GH16R1-R	CGACAACACGCAGACCTATG	
GH16R1-T7.F	T7 adapter-GCGAATCCAAATCACAGGCA	RNAi for AbGH16-2
GH16R1-T7.R	T7 adapter- CGACAACACGCAGAC-CTATG	
GFP-F	GGTGATGCAACATACGG	RNAi (control)
GFP-R	TGGTTGTCTGGTAAAAG	
GFP-T7.F	T7 adapter- GGTGATGCAACAT-ACGG	
GFP-T7.R	T7 adapter- TGGTTGTCTGGTAAAAG	
